# Structures of the germline-specific Deadhead and thioredoxin T proteins from *Drosophila melanogaster* reveal unique features among thioredoxins

**DOI:** 10.1107/S2052252521000221

**Published:** 2021-02-11

**Authors:** Regina Freier, Eric Aragón, Błażej Bagiński, Radoslaw Pluta, Pau Martin-Malpartida, Lidia Ruiz, Miriam Condeminas, Cayetano Gonzalez, Maria J. Macias

**Affiliations:** a Institute for Research in Biomedicine, The Barcelona Institute of Science and Technology, Baldiri Reixac 10, 08028 Barcelona, Spain; b ICREA, Passeig Lluís Companys 23, 08010 Barcelona, Spain

**Keywords:** thioredoxin structures, Schizophora, Deadhead, thioredoxin T, oxidoreductases, human diseases, crop plagues

## Abstract

Thioredoxins (Trxs) are ubiquitous enzymes that regulate the redox state in cells. In *Drosophila*, there are two germline-specific Trxs, Deadhead (Dhd) and thioredoxin T (TrxT), that belong to the lethal(3)malignant brain tumor signature genes and to the ‘survival network’ of genes that mediate the cellular response to DNA damage. Dhd is a maternal protein required for early embryogenesis that promotes protamine–histone exchange in fertilized eggs and midblastula transition. TrxT is testis-specific and associates with the lampbrush loops of the Y chromosome. Here, the first structures of Dhd and TrxT are presented, unveiling new features of these two thioredoxins. Dhd has positively charged patches on its surface, in contrast to the negatively charged surfaces commonly found in most Trxs. This distinctive charge distribution helps to define initial encounter complexes with DNA/RNA that will lead to final specific interactions with cofactors to promote chromatin remodeling. TrxT contains a C-terminal extension, which is mostly unstructured and highly flexible, that wraps the conserved core through a closed conformation. It is believed that these new structures can guide future work aimed at understanding embryo development and redox homeostasis in *Drosophila*. Moreover, due to their restricted presence in Schizophora (a section of the true flies), these structures can help in the design of small-molecular binders to modulate native redox homeostasis, thereby providing new applications for the control of plagues that cause human diseases and/or bring about economic losses by damaging crop production.

## Abstract 2

The first structures of the *Drosophila* germline-specific thioredoxins Deadhead (Dhd) and thioredoxin T (TrxT), which present unusual properties with respect to canonical Trxs, are reported. Dhd is highly positively charged, which facilitates nonspecific DNA binding to promote chromatin remodeling. TrxT contains a C-terminal extension, which is mostly unstructured and highly flexible, that modulates the redox activity of the protein. These structures will facilitate the virtual screening of small-molecule ligands and protein partners.

## 1. Introduction   

Thioredoxins (Trxs) are present in all living organisms and cellular compartments, and are therefore the most numerous subfamily of oxidoreductase enzymes in nature (Holmgren, 1985 ▸). In addition to their general role in controlling redox homeostasis in cells, Trxs participate in specific tasks, including the regulation of programmed cell death and transcription-factor activity and the modulation of inflammatory responses, and serve as growth factors (Collet & Messens, 2010 ▸). Trxs also contribute to protein folding and prevent protein aggregation. An example of this role is represented by the protein disulfide isomerase (PDI) family, which regulates protein misfolding by catalyzing the formation and breakage of disulfide bonds during protein synthesis (Wilkinson & Gilbert, 2004 ▸).

**Figure 1:**
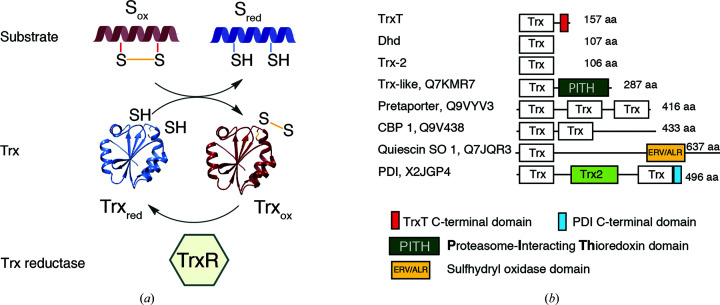

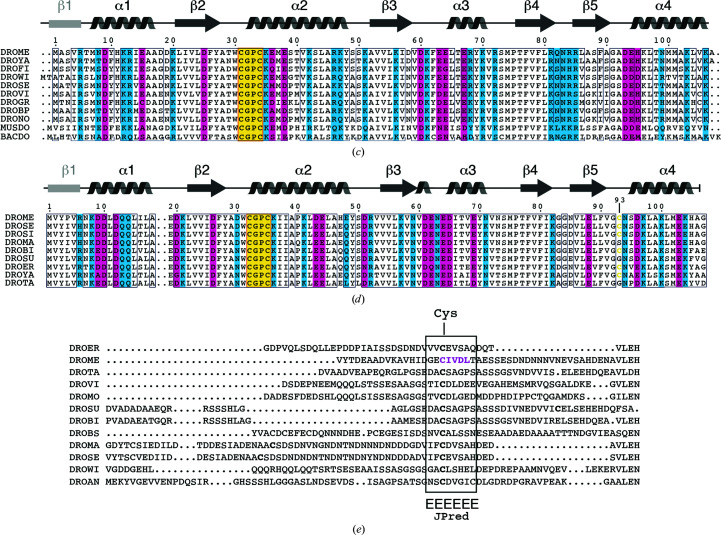
Redox mechanism and Trx proteins in *D. melanogaster*. (*a*) Schematic description of the Trx–TrxR redox mechanism adapted from Collet & Messens (2010 ▸). The redox cycle starts with a reduced form of Trx with the catalytic cysteine (Cys32) in the form of a thiolate [Supplementary Fig. S1(*a*)]. This state is stabilized by a hydrogen bond to the second cysteine of the motif (the Cys35 SH group). The thiolate is then able to form a transient intermolecular disulfide bond with the cysteine present in the oxidized substrate. The catalytic cycle ends when Cys35 in the enzyme attacks this intermolecular disulfide and forms a new intramolecular bond with Cys32, releasing the reduced substrate and the oxidized enzyme (Fomenko *et al.*, 2008 ▸). Trx recovers its initial state by the action of the Trx reductase (TrxR), which reduces Trx using NADPH/FAD as a source of reducing equivalents. (*b*) Trx-containing proteins in *D. melanogaster*. UniProt codes and domains are indicated. Abbreviations: CBP, calcium-binding protein; PDI, protein disulfide isomerase; SO, sulfhydryl oxidase. The ioelectric points for the different Trx domains are as follows: 6.5 for Q7KMR7, 5.0 for Q9VYV3 (three domains), 6.6 for Q9V438 (two domains), 4.9 for Q7JQR3 and 5.1 for X2JGP4 (two domains). A sequence alignment of these domains is shown in Supplementary Fig. S1(*b*). (*c*) Alignment of selected Dhd protein sequences. An extended version of this alignment is shown in Supplementary Fig. S1(*d*). The species are named with acronyms. RefSeq codes and species are given in Supplementary Table S1. The catalytic region is indicated with a yellow box and conserved positively and negatively charged residues are highlighted as blue and purple bars, respectively. Secondary-structure elements based on the *D. melanogaster* structure determined in this work are shown above the alignment. To facilitate comparison with other Trx structures, the β1 strand is indicated in gray. (*d*) Alignment of selected TrxT proteins. An extended version of this alignment is shown in Supplementary Fig. S1(*e*). Names and RefSeq codes are given in Supplementary Table S2. Colors are as in (*c*). (*e*) Sequence comparison of the TrxT C-terminal domain. The boxed region indicates the cysteine-containing motif predicted to adopt an extended conformation by *JPred* (Drozdetskiy *et al.*, 2015 ▸). The sequence that forms a disulfide bond with Cys93 and adopts an extended conformation in the crystals is indicated in pink.

Trxs share a conserved catalytic motif, with two conserved cysteine residues embedded in a canonical fold consisting of five β-strands and four α-helices. The catalytic motif (Cys-*X*-*X*-Cys) is located at the beginning of the second helix and is partially exposed to facilitate access to substrates [the structure of Trx-2 (Wahl *et al.*, 2005 ▸) is depicted as an example in Supplementary Fig. S1(*a*)]. The redox mechanism modulated by Trxs is a coordinated reaction in which a substrate protein and the thioredoxin/thioredoxin reductase system (Trx/TrxR) act in an orchestrated manner [schematically represented in Fig. 1 ▸(*a*)] (Collet & Messens, 2010 ▸). Of note, in humans the reduction of noncatalytic disulfide bonds that are present in Trxs is normally carried out by glutathione reductase proteins, but these proteins are absent in *Drosophila* species.

In *D. melanogaster* there are three main Trxs (https://flybase.org/) and several other proteins that contain Trx domains [Fig. 1 ▸(*b*), Supplementary Fig. S1(*b*)]. Among the specific Trxs, Trx-2 (also known as Dm Trx, and similar to human Trx1) is a non-essential protein that is widely distributed in all cellular compartments. The remaining two Trxs are the female germline-specific Deadhead (Dhd) protein and the male germline-specific TrxT, which have highly specific distributions and functional roles (Svensson *et al.*, 2003 ▸). Both TrxT and Dhd belong to the lethal(3)malignant brain tumor signature genes (Rossi *et al.*, 2017 ▸; Janic *et al.*, 2010 ▸). They have also been identified as part of the ‘survival network’ of genes that mediate the cellular response to DNA damage induced by the alkylating agent methyl methanesulfonate (MMS; Ravi *et al.*, 2009 ▸). TrxT has exclusive functions, such as its association with the Y chromosome lampbrush loops (Svensson *et al.*, 2003 ▸), whereas Dhd is essential for *Drosophila* embryo development. For instance, *dhd* ^−^ mutant oocytes show meiotic defects (Emelyanov & Fyodorov, 2016 ▸). Dhd is also required for early embryogenesis and metabolic remodeling, and it participates in the redox control of protamines (Ubbink, 2009 ▸) and in sperm chromatin remodeling *in vivo* (Rathke *et al.*, 2014 ▸; Tirmarche *et al.*, 2016 ▸). All of these roles are exclusive to Dhd, since the ubiquitous Trx-2 cannot recognize these substrates (Petrova *et al.*, 2018 ▸). Moreover, Dhd plays crucial roles in the oocyte-to-embryo transition, where it reduces and modulates the activity of ribosomal and RNA-binding proteins, as well as that of the histone de­methylase NO66 (Petrova *et al.*, 2018 ▸). Recently, the transcriptional regulation of Dhd has been reported to be modulated by the lysine-specific demethylase 5 (KDM5), a potent chromatin re­modeler during female gametogenesis (Torres-Campana *et al.*, 2020 ▸).

To identify the key features that distinguish the functions of the Dhd and TrxT proteins from that of Trx-2 in *Drosophila*, we turned our attention to studying the structures of the germline-specific TrxT and Dhd. Using X-ray crystallography and NMR spectroscopy, we found that both proteins display specific structural properties, thereby illustrating the versa­tility of the Trx fold to fine-tune its function. The information provided by these structures may guide future work aimed at understanding how redox inputs modulate the initial steps of embryo development in *Drosophila*, expanding the potential application of *Drosophila* as a model organism for studying redox regulation. We also observed that the Dhd and TrxT proteins are exclusively present in Schizophora (a section of the true flies), some species of which cause plagues that lead to human diseases and/or damage fruit and vegetable production. Since these proteins are absent in other insects, the structures determined here may represent the first step towards the design of molecular inhibitors using a structure-based approach to target specific plagues affecting the health and economies of many countries worldwide.

## 2. Methods   

### 2.1. Protein expression and purification   

The sequences of Dhd, TrxT and TrxT_ΔC (amino acids 1–111) from *D. melanogaster* were amplified from genomic DNA and cloned into the pOPINF expression vector. The constructs contained an N-terminal His_10_ tag followed by a 3C protease cleavage site. All clones were confirmed by DNA sequencing. All protein constructs were expressed in *Escherichia coli* BL21 (DE3) Rosetta cells following standard procedures. Unlabeled samples were prepared using Luria broth (Melford), and minimal medium M9 with ^15^NH_4_Cl and/or d-(^13^C)-glucose (Cambridge Isotope Laboratories) was used to prepare the labeled samples (Marley *et al.*, 2001 ▸). The cells were cultured at 37°C to reach an OD_600_ of 0.8–1.0. After induction with isopropyl β-d-1-thiogalactopyranoside (final concentration of 0.5 m*M*) and overnight expression at 20°C, the bacterial cultures were centrifuged and the cells were lysed using an EmulsiFlex-C5 (Avestin) or a Vibra-Cell (Sonics) in the presence of lysozyme and DNase I in phosphate-buffered saline pH 7.5. The soluble supernatants were purified by nickel-affinity chromatography (HiTrap Chelating HP column, GE Healthcare Life Sciences) using an NGC Quest 10 Plus Chromatography System (Bio-Rad) as described previously (Aragón *et al.*, 2019 ▸; Guca *et al.*, 2018 ▸; Martin-Malpartida *et al.*, 2017 ▸). Eluted proteins were digested with 3C protease at room temperature and further purified by size-exclusion chromatography on HiLoad Superdex 75 16/60 prep-grade columns (GE Healthcare) equilibrated with 10 m*M* Tris–HCl pH 7.5, 100 m*M* NaCl. For crystallography, the last step of purification was performed using 20 m*M* Tris–HCl pH 7.5, 100 m*M* NaCl, 2 m*M* ZnCl_2_. The purity of the recombinant proteins was greater than 95% as shown by mass-spectrometric analysis.

### 2.2. Sequence identification and clustering   

Dhd and TrxT sequences were retrieved using the protein *BLAST* (*BlastP*) search on the NCBI *BLAST* (*Basic Local Alignment Search Tool*) server, restricted to the non­redundant database and to dipteran insects. The search was first limited to highly conserved sequences using the Dm TrxT sequence as the query. Using the guide trees generated with the alignments, we clustered the different Trx-containing proteins. Each cluster was manually inspected for outliers and realigned using *Clustal Omega* (Sievers & Higgins, 2018 ▸), and the clusters containing the *D. melanogaster* TrxT and Dhd sequences were selected. As previously noted, Trx-2 showed the highest sequence similarity to other hits detected in insect species [Supplementary Fig. S1(*f*)], thus suggesting that Trx-2 is probably the ancestral Trx protein in Diptera and that TrxT and Dhd are the result of duplication events after the separation of Brachycera and Nematocera (Svensson *et al.*, 2007 ▸). TrxT sequences belonging to the subgenus *Sophophora* (specifically the subgroups *Melanogaster* and *Suzukii*, as well as the subgroup *Pseudoobscura*) contain a third cysteine residue at position 93. Accession numbers and names are collected in Supplementary Tables S1–S3.

*ESPript* 3.0 (Robert & Gouet, 2014 ▸) and *BoxShade* (https://embnet.vital-it.ch/software/BOX_form.html) were used to generate the figures as indicated in the figure legends. Secondary structure was predicted using the *JPred* v4 (Drozdetskiy *et al.*, 2015 ▸) and *PrDOS* (Ishida & Kinoshita, 2007 ▸) servers.

### 2.3. Differential scanning calorimetry   

Experiments were performed in a StepOnePlus Real-Time PCR System (Applied Biosystems). The assay was performed in 96-well plates (MicroAmp Fast 96-Well Reaction Plate, Applied Biosystems) with a total volume of 25 µl for each reaction. For stability screening, Slice pH (Hampton Research) was used. For additive screening (0–10 m*M* DTT, 0–10 m*M* TCEP, 0–10 m*M* ZnCl_2_, CaCl_2_, MgCl_2_), individual melting curves were acquired in triplicate and repeated twice. For each condition, the final protein concentration was 10 µ*M*. SYPRO Orange Dye (Sigma) was used at 60× dilution starting from a 5000× stock solution. Plates were sealed with optical quality sealing tape (PlateMax). Samples were equilibrated for 20 min and were analyzed using a linear gradient from 25 to 95°C in increments of 1°C min^−1^, recording the SYPRO Orange fluorescence throughout the gradient.

### 2.4. NMR experiments   

NMR data were recorded on a Bruker Avance III 600 MHz spectrometer equipped with a quadruple (^1^H, ^13^C, ^15^N, ^31^P) resonance cryogenic probe head and a *z* pulsed-field gradient unit at 298 K. Triple-resonance experiments were performed to obtain the backbone assignments of TrxT and Dhd using the *NMRlib* 2.0 package (Favier & Brutscher, 2019 ▸). Due to amino-acid repetitions in the TrxT sequence, obtaining the sequence-specific resonance assignment required the combination of standard backbone triple-resonance experiments (Favier & Brutscher, 2019 ▸) with site-specific amino-acid-type information using iHADAMAC experiments (Feuerstein *et al.*, 2012 ▸). Specific proline backbone assignment was assisted by dedicated experiments (Bottomley *et al.*, 1999 ▸). The assignment allowed us to identify 93 of the 107 residues present in Dhd and 123 of the 157 residues present in TrxT, including 48 of 51 residues located in the flexible C-terminal domain (amino acids 106–157). The data were processed using *NMRPipe* (Delaglio *et al.*, 1995 ▸) and were assigned using *Cara* (http://www.bionmr.com/).

Heteronuclear ^1^H–^15^N nuclear Overhauser enhancement (NOE), ^15^N transverse relaxation time (*T*_2_) and ^15^N longitudinal relaxation time (*T*_1_) spectra were acquired using pulse sequences described in the literature (Farrow *et al.*, 1994 ▸). ^1^H–^15^N NOE measurements were acquired using interleaved 2D heteronuclear single quantum-correlation spectroscopy (HSQC), with and without ^1^H saturation, with 256 ^15^N points and 24 (TrxT) or 40 (Dhd) scans per *T*_1_ increment. ^1^H saturation was implemented using a 120° ^1^H pulse train with 5 ms intervals. For *T*_2_ measurements, a series of experiments were performed with nine relaxation delays (0, 17, 34, 51, 68, 102, 136, 170 and 238 ms) using the ^15^N Carr–Purcell–Meiboom–Gill pulse train (Farrow *et al.*, 1994 ▸).

For *T*_1_ measurements, a series of experiments were conducted with 12 relaxation delays (20, 50, 110, 160, 270, 430, 540, 700, 860, 1080, 1400 and 1720 ms). A series of ^1^H off-resonance 180° pulses were applied at 5 ms intervals to suppress cross-correlation during the relaxation delay.

The raw data were processed and analyzed using the *TopSpin* 3.5 software (Bruker BioSpin). *T*_1_ and *T*_2_ relaxation times were calculated by nonlinear least-square fits of signal decays to an exponential decay function, *S*/*S*_0_ = exp(−*t*/*T*_1,2_) (Farrow *et al.*, 1994 ▸). τ_c_ values were calculated using the Stokes equation (Kay *et al.*, 1989 ▸),
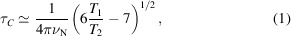
where ν_N_ is the ^15^N frequency in Hz.

^1^H–^15^N heteronuclear NOE values and ^15^N relaxation rates were used to estimate the flexibility of the proteins.

### 2.5. Crystallization   

TrxT was concentrated to 15 mg ml^−1^ in 10 m*M* Tris pH 7.5, 100 m*M* NaCl, 5 m*M* ZnCl_2_. Screenings and optimizations were prepared by mixing 100 nl protein solution and 100 nl reservoir solution in 96-well plates. Crystals were grown by sitting-drop vapor diffusion at 20°C. Crystals of TrxT were obtained in 15.0%(*w*/*v*) PEG 4000, 0.2 *M* potassium bromide. The Dhd sample was concentrated to 10 mg ml^−1^ in 20 m*M* Tris pH 7.2, 100 m*M* NaCl, 5 m*M* TCEP. Crystals were grown by sitting-drop vapor diffusion at 20°C. Screenings were prepared in three-drop 96-well plates by mixing protein and reservoir solution in 2:1, 1:1 and 1:2 ratios with final volumes of 300 nl. The best diffracting crystal was obtained in a drop comprising 200 nl protein sample and 100 nl reservoir solution consisting of 0.1 *M* sodium citrate pH 5.0, 3.2 *M* ammonium sulfate. It was cryoprotected by manual transfer to 0.1 *M* sodium citrate pH 5.0, 3.6 *M* ammonium sulfate.

### 2.6. Data collection and structure determination   

Diffraction data for TrxT were recorded on beamlines ID23-1 and ID23-2 at the European Synchrotron Radiation Facility (ESRF) and data for Dhd were recorded on the BL13-XALOC beamline at the ALBA Synchrotron Light Facility, Barcelona, Spain. Diffraction data were processed with *MOSFLM* and *XDS*, and were scaled and merged with *SCALA*, either alone or with *autoPROC* (Vonrhein *et al.*, 2011 ▸). Anisotropy correction for the Dhd data was applied using *STARANISO* (Tickle *et al.*, 2018 ▸). Initial phases were obtained by molecular replacement using *Phaser* (McCoy *et al.*, 2007 ▸) from the *CCP*4 suite (Murshudov *et al.*, 2011 ▸) with Trx-2 from *D. melanogaster* (PDB entry 1xwa; Wahl *et al.*, 2005 ▸) as a search model. *REFMAC* and *Phenix* (Liebschner *et al.*, 2019 ▸; Winn *et al.*, 2011 ▸) were used for refinement, and *Coot* (Emsley *et al.*, 2010 ▸) was used for manual improvement of the models. Figures were generated with *Coot* and *UCSF Chimera* (Pettersen *et al.*, 2004 ▸).

### 2.7. Thioredoxin activity assay   

To test the activity of wild-type TrxT in comparison to a shorter construct without the C-terminal extension (the TrxT_ΔC construct), we used a commercial kit for assaying mammalian Trx1 using a 96-well microplate format (IMCO Corporation, catalog No. FkTRX-02-V2). This method is based on the reduction of eosin-labeled insulin disulfides by Trx, with TrxR and NADPH as the ultimate electron donors. The emission at 545 nm after the excitation of eosin-labeled insulin at 520 nm was recorded for 30 min or up to 60 min. The assay was carried out using the following protocol. Briefly, Trx1 was diluted to a final concentration of 12 µg ml^−1^ (1 µ*M*), and 5 µl of freshly prepared β-NADPH solution was added and incubated for 30 min. Before the emission was recorded at 545 nm, 20 µl of the fluorescent substrate was added to all wells to start the reaction. The increasing fluorescence intensity over time of the reaction was calculated within a linear range to obtain a standard curve for human Trx1 activity and was then repeated for TrxT and Dhd. To test the activity of wild-type TrxT and TrxT without the C-terminus (residues 1–111), each construct was freshly produced and the concentration was set to 1 µ*M* (as determined by a NanoDrop and controlled by SDS–PAGE). The assay was repeated with two different batches of fresh protein.

### 2.8. Electrophoretic mobility-shift assay   

Cy5-labeled DNAs (purified by HPLC) were purchased from Metabion AG, Condalab, Spain. DNA duplexes were annealed using complementary DNAs. DNAs were mixed at equimolar concentrations (1 m*M*) in 20 m*M* Tris pH 7.0, 10 m*M* NaCl, heated at 90°C for 3 min and cooled to room temperature. DNA–protein binding reactions were carried out for 15 min at 4°C in 10 µl binding buffer (20 m*M* Tris pH 7.5, 100 m*M* NaCl, 2 m*M* TCEP). Prior to loading, samples were further diluted to 20 µl by adding Orange G loading dye (6×: 0.12 g Orange G in 100 ml 30% glycerol). For the reactions, a constant concentration of Cy5-labeled dsDNA of 7.5 n*M* was incubated with increasing concentrations of protein. Electrophoresis was performed in nondenaturing 6.0% 1.5 mm polyacrylamide gels prepared using 30% 19:1 acrylamide:bisacrylamide solution (Bio-Rad). The gels were pre-run to remove traces of ammonium persulfate at 80 V for 30 min and samples were run for 50 min in 1× TG buffer (25 m*M* Tris pH 8.4, 192 m*M* glycine) at 110 V at 4°C. The gels were exposed to a Typhoon imager (GE Healthcare) using a wavelength of 678/694 nm (excitation/emission maximum) for the Cy5 fluoro­phore.

## 3. Results   

### 3.1. Sequence comparison of *Drosophila* Trx proteins: specific features of Dhd and TrxT   

A comparison of *D. melanogaster* Trx sequences with those of vertebrate proteins revealed substantial differences in residues located at the N-terminus and also in the second half of the protein. These regions are highly conserved in mammals but are more variable in other vertebrates and in insects. As expected, the active site is highly conserved in all organisms [Supplementary Fig. S1(*c*) and Supplementary Table S1].

We used *Psi-BLAST* and the EMBL–EBI search tools to retrieve distantly related Dhd and TrxT proteins in recently sequenced invertebrates (see Section 2; Madeira *et al.*, 2019 ▸). The available data suggest that Dhd and TrxT are present in Schizophora (a section of the true flies) but absent in all other insects. Representative sequences correspond to the sub­sections Calyptratae (*Musca domestica*) and Acalyptratae (superfamilies Ephydroidea and Tephritoidea), and many members of the genus *Drosophila*.

Comparison of the Dhd sequences reveals the presence of abundant and conserved lysine and arginine residues (reflected by isoelectric point values of higher than 8) which are absent in the Trx-2 and TrxT sequences [Fig. 1 ▸(*c*), Supplementary Fig. S1(*e*) and Supplementary Table S2].

With respect to TrxT, the most obvious difference is the presence of a highly divergent C-terminal domain. This domain is variable in length and sequence, ranging from 33 residues in *Bactrocera oleae* (olive fruit fly) to 73 residues in *D. ananassae*. This C-terminal domain contains many negatively charged residues and one or two additional cysteines, but lacks the conserved hydrophobic residues that are often present in folded structures [Figs. 1 ▸(*d*) and 1 ▸(*e*), Supplementary Fig. S1(*e*) and Supplementary Table S3]. In fact, secondary-structure predictions of the C-terminal domain using *JPred* v4 (Drozdetskiy *et al.*, 2015 ▸) and the protein disorder-prediction system *PrDos* (Ishida & Kinoshita, 2007 ▸) only identified a short region with low disorder propensity surrounding one of the semiconserved cysteine residues [Fig. 1 ▸(*e*)].

### 3.2. Biophysical characterization of *D. melanogaster* Dhd and TrxT   

#### 3.2.1. Thermal stability of Dhd, TrxT and TrxT_ΔC   

**Figure 2:**
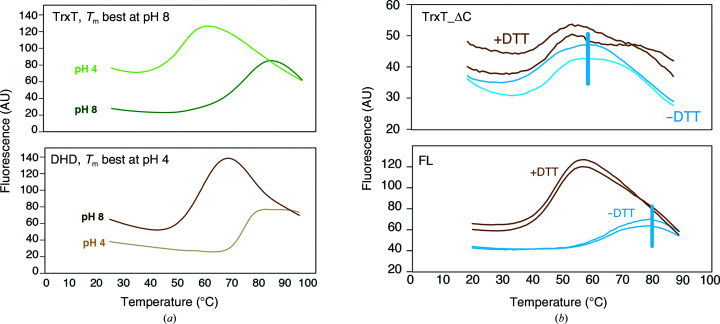

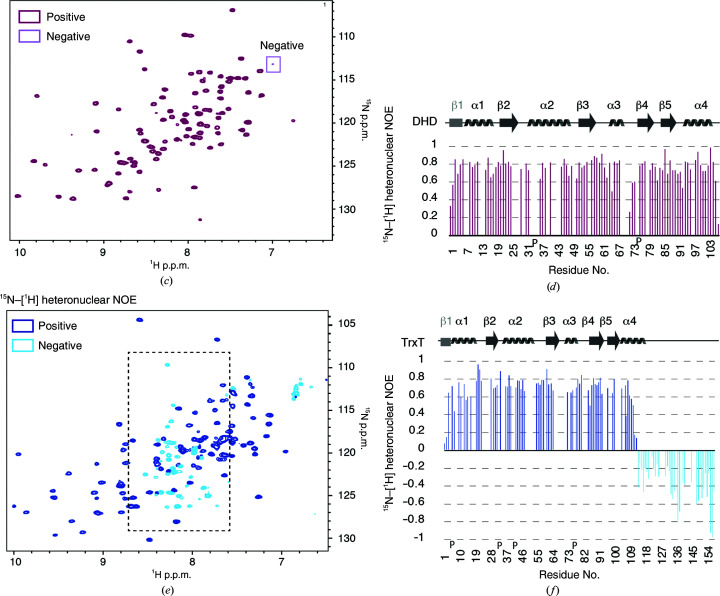
Stability and flexibility of TrxT and Dhd. (*a*) Thermal shift assay of TrxT and Dhd after incubation of natively folded proteins with SYPRO Orange dye in a 96-well PCR plate at two different pH values. As the proteins unfold with temperature, the SYPRO Orange fluorescence emission increases. TrxT is thermally stable with a *T*_m_ of >70°C in alkaline buffers, whereas acidic buffers are necessary for Dhd to reach the same stability. Values were obtained in triplicate and were collected under different conditions (Supplementary Table S2). (*b*) Thermal stability of full-length (FL) TrxT and TrxT_ΔC constructs in the presence or absence of DTT (duplicates). FL TrxT is ∼20°C more stable than the protein core. (*c*)^15^N–[^1^H] heteronuclear NOE experiment for Dhd in the absence of DTT. The same experiment with DTT is shown in Supplementary Fig. 2 ▸(*f*). (*d*) ^15^N–[^1^H] heteronuclear NOEs were measured as duplicates for Dhd (red). All values are positive, with the exception of a side-chain resonance. The missing bars correspond to prolines and residues for which amide resonances were not assigned. (*e*) ^15^N–[^1^H] heteronuclear NOE experiment of TrxT in the absence of DTT. Assignments corresponding to the flexible residues (all located in the C-terminal domain) are shown in Supplementary Fig. S2(*d*). The same experiment with DTT is shown in Supplementary Fig. S2(*e*). (*f*) ^15^N–[^1^H] heteronuclear NOEs were measured as duplicates for TrxT. Negative values are characteristic of highly flexible regions. Positive and negative values are shown in royal blue and light blue as in (*c*). The missing bars correspond to prolines and residues for which amide resonances were not assigned.

We purified the recombinant Dm Dhd and TrxT proteins and found that these proteins are stable at opposite pH values: TrxT is thermally stable in alkaline buffers with a *T*_m_ of >70°C, whereas acidic buffers are necessary for Dhd to reach the same stability [Fig. 2 ▸(*a*), Supplementary Table S4]. Both Dhd and TrxT were well folded, as revealed by 2D ^1^H–^15^N TROSY experiments [Supplementary Fig. S2(*a*)]. Addition of DTT to Dhd did not affect the thermal stability of the protein; however, the *T*_m_ value was reduced by approximately 20°C in the case of TrxT [Fig. 2 ▸(*b*)], suggesting the presence of an additional disulfide bond outside the protein core that is important for the stability of TrxT. To test this hypothesis, we expressed an additional TrxT construct without the C-terminal region (TrxT_ΔC; residues 1–111). As expected, the TrxT_ΔC protein was 20°C less stable than the wild-type protein and displayed progressive unfolding and aggregation under experimental conditions in which TrxT was well folded (monitored through a series of 2D ^1^H–^15^N TROSY acquired over 2 h; Supplementary Figs. S2(*b*) and S2(*c*)].

#### 3.2.2. NMR relaxation experiments confirm that the Dhd fold is compact and that the TrxT C-terminal extension is flexible   

Residues in flexible and/or well structured regions exhibit different internal motions, which can be identified through the analysis of backbone amide ^15^N relaxation parameters, including the ^15^N–[^1^H] heteronuclear NOE and ^15^N longitudinal (*T*_1_) and transverse (*T*_2_) values (Kosol *et al.*, 2013 ▸). Compact folds display positive ^15^N–[^1^H] heteronuclear NOE values, whereas flexible loops and disordered regions often display low or negative ^15^N–[^1^H] NOE values. In Dhd, all of the ^15^N–[^1^H] heteronuclear NOE peaks were positive, thus indicating that the Dhd fold is highly compact in solution. The *T*_1_ and *T*_2_ values revealed that the protein behaves as a monomer of ∼11 kDa [τ_c_ of 6.7 ns with *T*_1_ = 620 ms and *T*_2_ = 110 ms; Figs. 2 ▸(*d*) and 2 ▸(*e*) and Supplementary Fig. S2(*d*)]. In TrxT, the ^15^N–[^1^H] NOE peaks for residues located in the protein core were positive and had a similar intensity to those of Dhd, whereas the residues in the C-terminal region (from Ala106 to His120) displayed significantly low values and the ^15^N–[^1^H] NOE values for the most C-terminal part of the domain were negative, indicating intermediate to fast internal motions that were faster than other residues assigned to the protein core [Figs. 2 ▸(*e*), 2 ▸(*f*) and Supplementary Figs. S2(*e*) and S2(*f*)]. Moreover, the chemical shift values (CSVs) of residues in this C-terminal region indicate the absence of secondary structure, which was corroborated by the absence of medium-range NOEs for the residues following Ala106 in the 3D ^15^N NOESY–HSQC spectrum. Together, these results indicate that the C-terminal domain is dynamic in solution. The overall *T*_1_ and *T*_2_ values (*T*_1_ = 800 ms, *T*_2_ = 67 ms) and correlation time, τ_c_ = 10 ns, agree with a monomeric and compact ∼17 kDa protein (the theoretical molecular weight was 17.5 kDa).

Focusing on the cysteine residues, we observed that the ^13^C CSVs for Cys93 and Cys125 indicate a mixture of redox states and conformations (30.4 and 34.2 p.p.m., respectively), suggesting the presence of a disulfide bond between Cys93 and Cys125 in at least half of the conformations (Sharma & Rajarathnam, 2000 ▸). Due to the monomeric behavior of the protein in solution, this disulfide bond is mostly intramolecular.

### 3.3. Structures of Dhd and TrxT   

We used X-ray crystallography for structural characterization of the Dhd and TrxT proteins. Attempts to obtain crystals of Dhd, TrxT and TrxT_ΔC were performed in the presence and absence of reducing agents (TCEP). However, TrxT crystals were only obtained in the absence of TCEP, whereas the best diffracting Dhd crystals were obtained in buffers containing TCEP. No crystals were obtained for the TrxT_ΔC construct.

**Figure 3:**
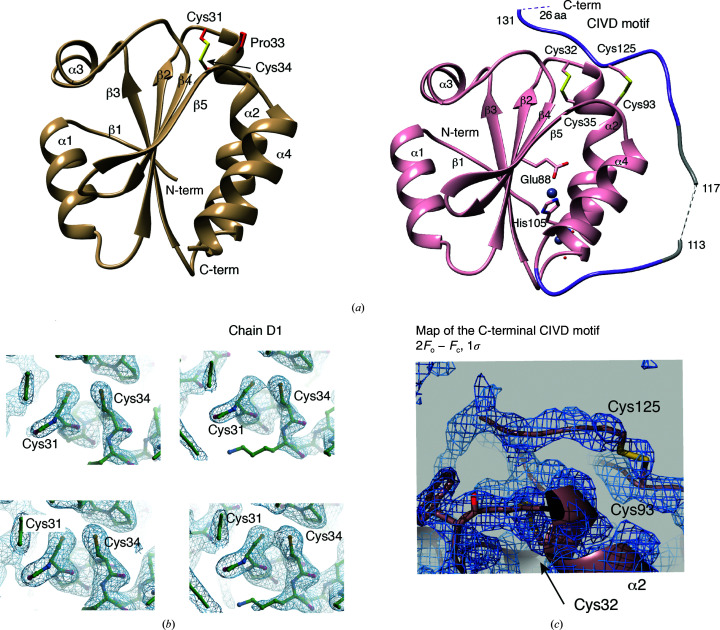
Crystal structures of *D. melanogaster* Dhd and TrxT. (*a*) Cartoon representation of the crystal structures of Dhd (left) and TrxT (right), showing the oxidized forms. Cys31/Cys32 and Cys34/35 in the catalytic motif are labeled. Secondary-structure elements are also indicated. For TrxT, part of the C-­terminal domain was connected to the Trx core domain by a disulfide bridge between Cys93 and Cys125. (*b*) Electron-density map of the Dhd active center including the pair of cysteines Cys31 and Cys34. Left: reduced chain *B*. Right: partially oxidized chain *D*. Top: 2*F*_o_ − *F* *c* electron-density map contoured at 2.0σ. Bottom: 2*F*_o_ − *F* *c* electron-density map contoured at 1.0σ. (*c*) The 2*F*_o_ − *F* *c* electron-density map for the bound C-terminal fragment contoured at 1.0σ.

The asymmetric units of TrxT and Dhd contain one and four monomers, respectively [Fig. 3 ▸(*a*), Supplementary Fig. S3(*a*) and Table 1 ▸]. In general, the Trx fold is defined by a core structure that contains three α-helices and four β-strands, although most Trx structures fold as four α-helices and five β-strands (Collet & Messens, 2010 ▸). *Drosophila* Dhd, TrxT and Trx-2 (previously determined; Wahl *et al.*, 2005 ▸) are intermediate between these two characteristic folds, displaying four α-helices (as in all Trx structures) but only four β-strands (β2–β5). The N-terminal region, which corresponds to the β1 strand in other Trx structures, is only stabilized by a single set of hydrogen bonds with β3, and it is not defined as a proper β1 strand [Fig. 3 ▸(*a*)]. The β-strands of the C-terminal region (β2, β4 and β5) run antiparallel, whereas β2 and β3 are oriented parallel to one another [Fig. 3 ▸(*a*)]. In both the TrxT and Dhd structures, the active sites (Cys-Gly-Pro-Cys) are located between the β2 strand and the N-terminal part of the α2 helix, which is slightly curved due to the bend caused by the presence of a proline (TrxT) or a serine (Dhd) residue in the middle part of the helix. Probably due to the buffer conditions, the active sites are oxidized in the case of TrxT and reduced in the case of Dhd, although in this case the electron density for one of the four monomers (chain *D*) indicates the presence of the catalytic Cys–Cys disulfide bridge in equilibrium with the reduced form [Figs. 3 ▸(*a*) and 3 ▸(*b*)]. Moreover, in the case of TrxT the crystals contain two TrxT molecules engaged with a symmetry-related neighbor stabilized through the coordination of a Zn atom (which was present in the crystallization condition). The zinc is bound between Asp65 and Glu69 from monomer *A* and His105 and Glu88 from monomer *B* [Supplementary Fig. S3(*b*)]. We found that the C-terminal domain contributes to the structure of the protein through the presence of a closed conformation. The 2*F*_o_ − *F*_c_ density plotted at 1σ showed that Cys93 in TrxT, located in the loop connecting β5 and α4, forms a covalent bond to Cys125 of the C-terminal domain in 70% of the molecules, preventing oligomerization via disulfide bonds between monomers [Fig. 3 ▸(*c*)]. These results confirm that the C-terminal domain is part of this specific Trx structure, which is in agreement with the correlation times and monomeric behavior determined by NMR. This close conformation also explains the decrease in stability displayed by the TrxT_ΔC construct or upon the addition of DTT in thermal denaturation assays. The regions connecting Gly107 to Asp111 and His120 to Cys125 were also traceable, but unfortunately the fragment connecting Asp111 to His120 and the last 26 amino acids were not visible in the density, which is also in agreement with the rapid motions and negative ^15^N–[^1^H] NOE values measured for these residues.

### 3.4. Comparison to other Trx structures   

Apart from the differences at the secondary-structure level (the absence of the β1 strand and the presence of the C-terminal domain in TrxT), the overall core structure of Dhd and TrxT is well conserved with respect to other Trxs. One of the differences is found at Pro76, which adopts a *trans* configuration in Dhd, TrxT and Trx-2 and not *cis* as reported for other Trxs (Collet & Messens, 2010 ▸). When these new structures are superimposed on that of Trx-2 (Wahl *et al.*, 2005 ▸), we obtained root-mean-square deviation (r.m.s.d.) values of ∼0.9 Å for Dhd and ∼1.2 Å for TrxT (for all residues in the core, including loops).

**Figure 4:**
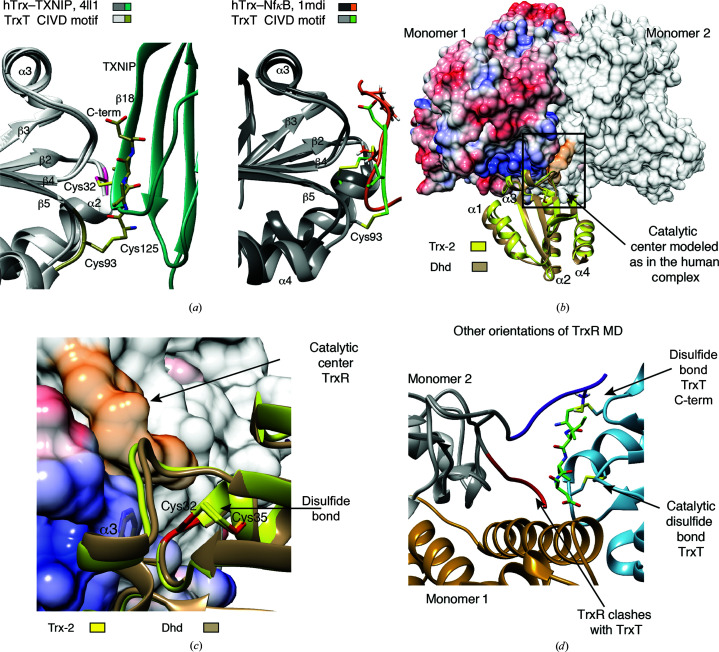

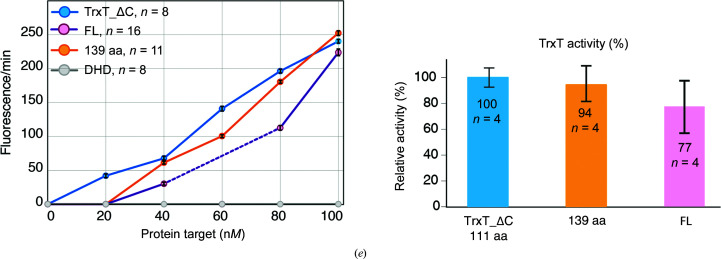
Comparison with other Trx protein complexes. The C-terminal fragment modulates the stability and redox activity of TrxT. (*a*) Comparison of the overall structure of TrxT with those of human Trx in complex with TXNIP (left; PDB entry 4ll1) and human Trx in complex with NFκB (right; PDB entry 1mdi), using human Trx for the fitting. The C-terminal CIVD motif of TrxT (shown in chartreuse) occupies the same position as the Trx partners in the human Trx complex structures. Full molecules are shown in Supplementary Figs. S4(*a*) and S4(*b*). (*b*) Model of Trx-2 and Dhd structures docked to Dm TrxR as observed in the human TrxR–Trx complex. The catalytic center of TrxR is able to access the Cys32–Cys35 bond, shown in red, in both *Drosophila* thioredoxins (PDB entry 3qfa). (*c*) Close-up view of the interaction between the catalytic center of the reductase (colored orange and indicated with an arrow) and the oxidized forms of Trx-2 (yellow) and Dhd (tan) rotated 90° with respect to the view shown in (*b*). The catalytic Trx and Dhd cysteines are shown in red. The α3 helix of Dm Trx and Dhd that participates in direct contacts with the reductase is labeled. (*d*) Close-up view of the model of TrxT bound to Dm TrxR as depicted in Fig. 4 ▸(*b*). The catalytic center of TrxR as determined in the human complex cannot access the Cys32–Cys35 bond in TrxT due to the presence of the C-terminal motif attached to Cys93 in the core domain (both sites are indicated with arrows). The orientation shown in purple will allow the reduction of Cys93–Cys125 of the tail. Once this step is achieved, a second reaction can occur to reduce the Cys32–Cys35 bond. (*e*) Redox activity of TrxT constructs against an eosin-labeled insulin disulfide substrate using a commercial kit developed by IMCO Corporation. The activity of the full-length TrxT protein is slightly less than that of the totally processed C-terminal domain. However, the C-terminal domain contributes to the stability of the full-length protein, increasing the melting temperature by ∼20°C. Repetitions and error bars corresponding to the standard error are indicated.

We found a structural convergence between the structure of TrxT and those of Trx complexes with target proteins described in the literature. For instance, overlapping the structure of TrxT with that of the complex of human Trx and the thioredoxin-interacting protein (TXNIP; PDB entry 4ll1) [Fig. 4 ▸(*a*) and Supplementary Fig. S4(*a*)] showed that the C-terminal fragment of TrxT binds in a similar manner and within the same site as observed in the TXNIP–Trx complex (Hwang *et al.*, 2014 ▸). In fact, the TXNIP–Trx interaction serves to inhibit Trx redox activity by impairing access to the catalytic site of Trx (Hwang *et al.*, 2014 ▸). Although there is a parallelism in both structures, the interaction involves distinct cysteine residues: an intramolecular disulfide Cys93–Cys125 in TrxT versus the intermolecular disulfide between human Trx and TXNIP [Fig. 4 ▸(*a*), left]. A similar intermolecular interaction is also observed in the complex of human Trx with one of its substrates, the transcription factor NfκB, bound to the catalytic Cys32 (PDB entry 1mdi; Matthews *et al.*, 1992 ▸) [Fig. 4 ▸(*a*), right].

Another conserved characteristic of Dhd and TrxT with respect to other Trx proteins is the presence of a negatively charged surface patch in helix α3, which is required to interact with TrxR to recover the redox equilibrium (Rigobello & Bindoli, 2010 ▸). The available TrxR structures revealed that these proteins are mostly homodimeric enzymes in which the catalytic site, which is either a cysteine residue in insects or a selenocysteine residue in mammals, is located at the flexible C-terminus (Holmgren, 1985 ▸; Arnér & Holmgren, 2000 ▸; Powis *et al.*, 2000 ▸; Tamura & Stadtman, 1996 ▸).

Using the human Trx–TrxR complex as a template (PDB entry 3qfa; Eckenroth *et al.*, 2007 ▸), we docked Dhd or Trx-2 onto the surface of TrxR superposed with the human Trx protein. Both the Dhd and Trx-2 models bound to TrxR fitted well to the template [Fig. 4 ▸(*b*)], with the Cys32 side chains of the Dhd and Trx-2 proteins being accessible to the reductase. However, on docking TrxT in a similar manner the C-terminal domain blocks the approach of TrxR to Cys32 [Fig. 4 ▸(*c*)]. Since the catalytic loop of TrxR is flexible (Eckenroth *et al.*, 2007 ▸), we propose that a slight reorientation of this loop (as in our model) would permit a subtle variation of the mechanism, first reducing the Cys93–Cys125 bond, and thus promoting a transition from the closed to an open conformation, and then reducing the accessible Cys32–Cys35 [Figs. 4 ▸(*c*) and 4 ▸(*d*)].

### 3.5. The C-terminal domain of Dm TrxT modulates its redox activity *in vitro*   

To evaluate our hypothesis, we experimentally analyzed the activity of two TrxT constructs (full-length and TrxT_ΔC) towards an eosin-labeled insulin peptide using a commercial assay, which provides a mammalian TrxR and a human Trx as positive controls. Even using the human TrxR, which has a smaller reduction potential than that from *Drosophila* (Eckenroth *et al.*, 2007 ▸), we observed that the full-length TrxT construct reduced the eosin-labeled insulin substrate by approximately 80% compared with the short construct lacking the C-terminal extension. No effect was observed for Dhd under these experimental conditions, probably due to the basic pH recommended for the assay, which compromises the stability of Dhd [Fig. 4 ▸(*e*)]. Overall, these results underline the hypothesis that the C-terminal domain shields the active site of TrxT and modulates its redox activity. Certainly, further structural studies will be needed to clarify the precise mechanisms of these reactions.

### 3.6. The surface of Dhd is positively charged and facilitates the association of Dhd with DNA   

**Figure 5:**
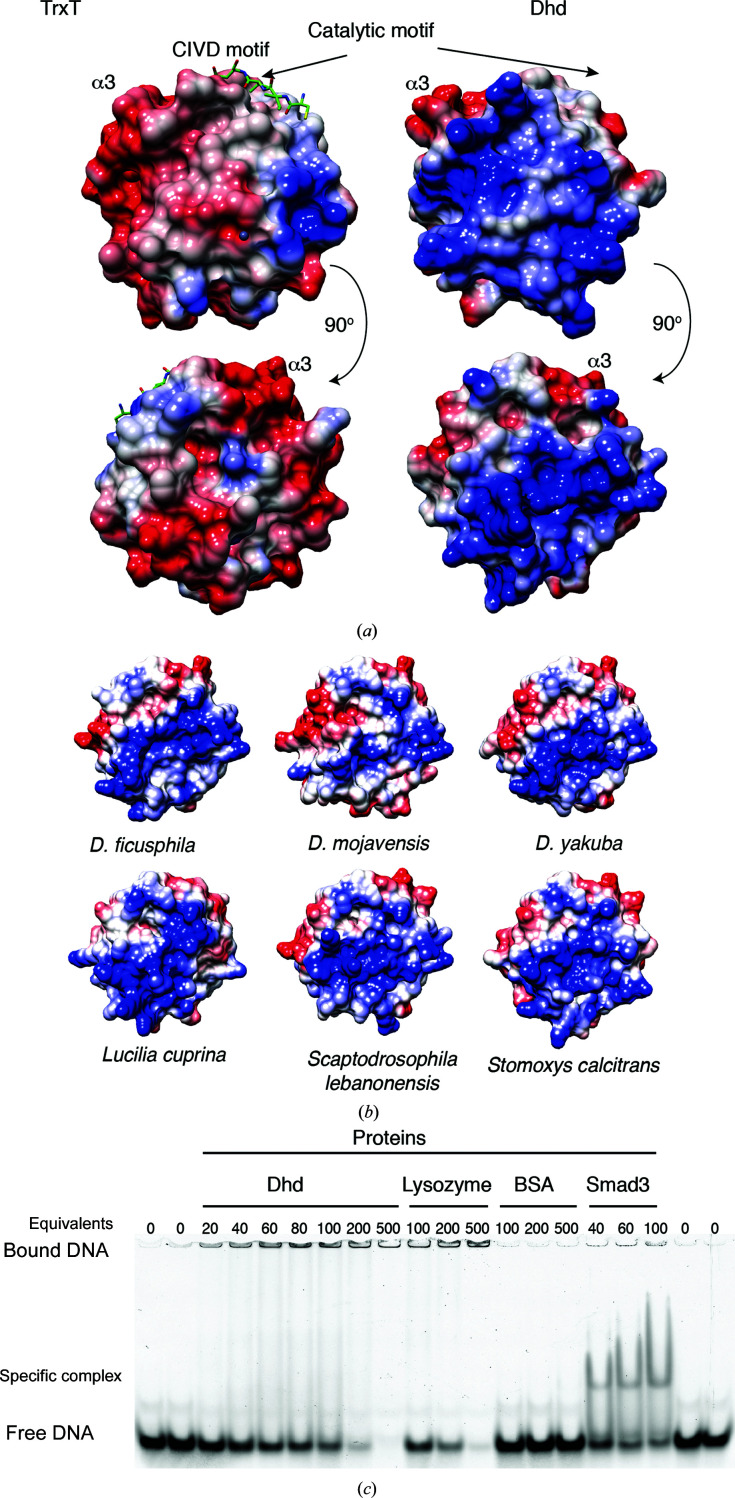
Charge distribution and DNA binding. (*a*) Surface-charge distribution for the TrxT (left) and Dhd (right) proteins. Both sides of the surface are displayed. The catalytic site, the α3 helix and the C-terminal CIVD motif of TrxT are indicated. (*b*) Surface-charge distribution of Dhd proteins generated using the Dm Dhd structure as a template. The molecules are oriented as in the top view in (*a*). The other side is represented in Supplementary Fig. S4(*c*). (*c*) The DNA-binding capacity of Dm Dhd compared with those of lysozyme, BSA and the MH1 domain of Smad3. Protein concentrations are indicated at the top of the gel. The DNA is at 7.5 n*M*. Dm Dhd and Smad3 interact with DNA at a 40 equivalents protein excess, although the interaction of Dhd is nonspecific whereas Smad3 binds to a single site in this DNA.

Eukaryotic Trxs frequently have negatively charged patches on their surfaces, as is the case for *D. melanogaster* Trx-2 and TrxT [Supplementary Fig. S1(*a*) and Fig. 5 ▸(*a*), left]. However, *D. melanogaster* Dhd does not follow this rule and presents an unusual positively charged surface [Fig. 5 ▸(*a*), right]. The presence of positively charged patches is often taken as an indication of membrane binding to phospholipids or as a protein–DNA/RNA binding patch. In fact, the known targets of Dhd include protamine proteins, ribosomes and ribosome-associated factors, thereby suggesting a role of this specific charge distribution in selecting protein and DNA/RNA partners (Petrova *et al.*, 2018 ▸).

The residues responsible for these patches are partially conserved in other Schizophora sequences [Fig. 1 ▸(*c*), Supplementary Fig. S1(*d*)]. The presence of these positively charged patches defines a specific feature distinguishing Dhd from other Trxs and also distinguishing individual Dhd proteins. We modeled six Dhd sequences onto the *D. melanogaster* Dhd structure and observed that the positively charged patches are likely to be present in other Dhd proteins [Supplementary Figs. S4(*b*) and S4(*c*)].

We have also explored whether these patches help Dhd proteins to associate with DNA in a non-sequence-specific manner using an electrophoretic mobility-shift assay (EMSA), as Dhd proteins are involved in the reduction of protamines and ribosomal proteins. For this purpose, we compared the interaction of Dhd with lysozyme (a highly positively charged protein that interacts with DNA in a non-sequence-specific manner; Steinrauf *et al.*, 1999 ▸; Lin *et al.*, 2009 ▸) and with the MH1 domain of Smad3 (an example of specific interaction; Macias *et al.*, 2015 ▸; Martin-Malpartida *et al.*, 2017 ▸). BSA was used as a negative control as a protein that does not interact with DNA. As indicated in the EMSA [Fig. 5 ▸(*c*)] both Dhd and lysozyme showed a super-shift of the DNA, whereas the Smad3 MH1 domain binds to DNA as a well defined interaction. In contrast, the presence of increasing concentrations of BSA did not alter the band corresponding to unbound DNA. Attempts to characterize the Dhd residues involved in nonspecific DNA binding using NMR were not successful because the protein aggregates in the presence of DNA (presumably because several DNA molecules decorate the protein surface and nucleate large protein–DNA complexes).

## 4. Discussion   

Many drug-discovery strategies for aging, anticancer and Parkinson therapies (Gonzalez, 2013 ▸) have taken advantage of model organisms such as *Drosophila* as cost-effective alternatives to mammalian cellular/animal systems. Trx reductase (TrxR) and Trxs are overexpressed in many tumor cells, the proliferation of which is dependent on a high supply of deoxyribonucleotides (Grogan *et al.*, 2000 ▸; Smart *et al.*, 2004 ▸). Hence, inhibition of the Trx/TrxR system has emerged as an attractive target for anticancer drugs to induce cell death (Bradshaw *et al.*, 2005 ▸; Richardson *et al.*, 2015 ▸). This approach is based on the conservation of many pathways in metazoans as well as on a good knowledge of the differences. The new structures revealed two main differences of these specific germline Trxs with respect to other previously characterized Trx structures. Whereas the Trx surfaces, including TrxT, are negatively charged, Dhd has extended positively charged patches. These areas are likely to be present in other structures of Schizophora species, as the arginine and lysine residues responsible for these patches are abundant in all Dhd proteins [Supplementary Figs. S1(*d*) and S1(*f*)]. By displaying a positively charged distribution (and not negative as in most Trxs), Dhd proteins would speed up the selection of specific redox targets during initial encounter complexes *in vivo* (Ubbink, 2009 ▸; Lagunas *et al.*, 2018 ▸). Among these redox reactions are the reduction of intermolecular disulfide bonds in *Drosophila* protamine oligomers to facilitate their eviction from DNA (Emelyanov & Fyodorov, 2016 ▸). To perform this function, Dhd proteins must get close to the protamine oligomers bound to DNA, and as we have observed in the DNA-binding assays this approach to DNA would be facilitated by charge complementarity (negative at the DNA backbone and positive at the Dhd surface). These differences in charge distribution probably explain why Dhd cannot be replaced by Trx-2 in *in vivo* assays. This charge complementarity would also favor the interaction with ribosomes and associated factors such as NO66, which is a lysine-specific demethylase that removes methyl groups from histone H3, and with the ribosome protein 8 (Rpl8) hydroxylase, as described previously (Wang *et al.*, 2015 ▸).

The second difference is related to the TrxT fold, which is composed of a highly conserved core and a variable C-terminal domain, with the latter being absent in most canonical Trxs. This extra domain is mostly unstructured, with a high ratio of negatively charged residues. In *D. melanogaster*, this C-terminal domain is attached to the protein core via a covalent link that stabilizes a closed conformation that partially covers the catalytic site. We observed that many TrxT proteins have a cysteine in the C-terminal domain, but only a third of them also have Cys93 conserved. The latter feature indicates that not all dipteran TrxT proteins can form a second disulfide bond as observed in *D. melanogaster* TrxT and that the C-terminal domains of other TrxTs might perhaps adopt different orientations. The high sequence variability of this region might also contribute as an additional switch to regulate TrxT–protein interactions in an almost species-specific manner.

Apart from the catalytic site, the presence of additional disulfide bonds has been observed in other Trxs, for example in human Trx1. In this case, this intramolecular bond involves Cys62 and Cys69 and inactivates the redox capacity of the protein (Du *et al.*, 2013 ▸). Compared with this inhibitory role in human Trx1, the formation of the additional disulfide bond in TrxT seems to have a mild effect on its function. However, this additional disulfide bond might provide a mechanism by which the stability of the protein can be increased not only *in vitro*, as we have characterized, but perhaps also *in vivo*.

Moreover, our results may have applications for the design of inhibitory molecules to reduce and control fly plagues by selecting the germline Trx proteins as targets. These plagues, such as those of black fly species that spread diseases such as river blindness in Africa and the Americas (World Health Organization), have an impact on human health. Others negatively affect the economies of many countries worldwide due to losses in fruit and vegetable production. In this context, the charge distribution of Dhd should drive the selection of molecular binders which differ from those preferentially selected by Trx-2 (present in many insects) and TrxT counterparts. The structures that we have determined will help to correlate binding effects with phenotypes during oxidative stress and redox signaling, separating germline-specific roles from general features of the common Trx-2 protein. This knowledge may also guide the docking of *Drosophila* protein partners described in the literature (Petrova *et al.*, 2018 ▸).

## 5. Data availability   

Atomic coordinates and electronic densities for the reported crystal structures have been deposited in the Protein Data bank under accession numbers 6zmu (Dhd) and 6z7o (TrxT). NMR chemical shifts have been deposited in the BMRB with IDs 50419 (Dhd) and 50420 (TrxT).

## 6. Related literature   

The following reference is cited in the supporting information for this article: Kozlowski (2016 ▸).

## Supplementary Material

PDB reference: Deadhead, 6zmu

PDB reference: thioredoxin T, 6z7o

Supplementary Tables and Figures. DOI: 10.1107/S2052252521000221/jt5053sup1.pdf

## Figures and Tables

**Table 1:** Data-collection and refinement statistics Values in parentheses are for the highest resolution shell.

	TrxT	Dhd
PDB entry	6z7o	6zmu
Resolution range	35.13–2.24 (2.32–2.24)	77.31–1.95 (2.05–1.97)†
Space group	*P*2_1_2_1_2_1_	*P*4_3_2_1_2
*a*, *b*, *c* (Å)	46.09, 49.31, 54.28	111.83, 111.83, 107.00
α, β, γ (°)	90, 90, 90	90, 90, 90
Total reflections	40618 (3817)	473364 (21602)
Unique reflections	4697 (457)	43714 (2187)
Multiplicity	8.0 (8.4)	10.8 (9.9)
Completeness (%)	99.9 (99.8)	90.0 (38.3)†
〈*I*/σ(*I*)〉	9.4 (2.2)	18.1 (1.5)
Wilson *B* factor (Å^2^)	39.06	44.68
*R*_merge_	0.18 (0.86)	0.07 (1.50)
*R*_meas_	0.19 (0.91)	0.07 (1.57)
CC_1/2_	0.99 (0.85)	1.00 (0.62)
*R*_work_/*R*_free_	0.20/0.23	0.19/0.22
No. of non-H atoms		
Total	904	3606
Macromolecules	872	3436
Ligands	2	76
No. of protein residues	110	423
R.m.s.d., bonds (Å)	0.010	0.86
R.m.s.d., angles (°)	0.93	0.99
Ramachandran favored (%)	98.1	97.8
Ramachandran allowed (%)	1.9	2.2
Ramachandran outliers (%)	0	0
Rotamer outliers (%)	0	3.2
Clashscore	9.18	6.95
Average *B* factor (Å^2^)		
Overall	40.64	39.07
Macromolecules	40.61	37.61
Ligands	45.00	97.88
Solvent	41.10	44.83

† Anisotropy correction by *STARANISO*/*autoPROC* with the CC_1/2_ criterion used for the resolution cutoff (Vonrhein *et al.*, 2011 ▸; Tickle *et al.*, 2018 ▸).
