# Supplementary material for: Structures of the germline-specific Deadhead and thioredoxin T proteins from Drosophila melanogaster reveal unique features among thioredoxins
Source: IUCrJ. 2021 Feb 11;8(Pt 2):281–94. doi: 10.1107/S2052252521000221 (PMC7924233; doi:10.1107/S2052252521000221)
Supplement: Supplementary file 1 [file m-08-00281-sup1.pdf]

IUCrJ

Volume 8 (2021)

Supporting information for article:

Structures of the germline-specific Deadhead and thioredoxin T proteins from *Drosophila melanogaster* reveal unique features among thioredoxins

Regina Freier, Eric Aragon, Blazej Baginski, Radoslaw Pluta, Pau Martin-Malpartida, Lidia Ruiz, Miriam Condeminas, Cayetano Gonzalez and Maria J. Macias

Table S1 Thioredoxin acronyms used in Supplementary-Figure S1.

Alignment name	Species	RefSeq
Trx1 - Human	*Homo sapiens*	AF313911.1
Trx1- Marmoset	*Callithrix jacchus*	AF353204.1
Trx1 - Mouse	*Mus musculus*	NP_035790
Trx1 - Blind mole rat	*Nannospalax galili*	XP_008852851
Trx1 - Horse	*Equus caballus*	NP_001075282
Trx1 - Arabian camel	*Camelus dromedarius*	XP_010977020
Trx - Sperm whale	*Physeter catodon*	XP_007130171
Trx - Dingo	*Canis lupus dingo*	XP_025288383
Trx - European Hedgehog	*Erinaceus europaeus*	XP_016049782
Trx - Egyptian fruit bat	*Rousettus aegyptiacus*	XP_015985530
Trx - Amur tiger	*Panthera tigris altaica*	XP_015393030
Trx - White Rhinoceros	*Ceratotherium simum simum*	XP_004423376
Trx- Chicken	*Gallus gallus*	NP_990784
Trx - Giant devil catfish	*Bagarius yarrelli*	TSK22521
Trx - Northern pike Lucius	*Esox lucius*	NP_001290895
Trx - Torafugu	*Takifugu rubripes*	XP_003978603
Trx - Xenopus Tropicalis A	*Xenopus tropicalis*	XP_002940306
Trx - Xenopus Tropicalis B	*Xenopus tropicalis*	XP_031746215
TrxT - D. melanogaster	*Drosophila melanogaster*	AAF46018
Trx2 - D. melanogaster	*Drosophila melanogaster*	AAN10700
Dhd/Trx1 - D. melanogaster	*Drosophila melanogaster*	AHN59353

Table S2 Dhd acronyms and entries used in the alignments

Acronym	Species	RefSeq	Isoelectric Point
DROME	*Drosophila melanogaster*	NP_001284882.1	8.7
DROYA	*Drosophila yakuba*	XP_002100027.1	8.7
DROFI	*Drosophila ficusphila*	XP_017051920.1	8.6
DROWI	*Drosophila willistoni*	XP_002071506.1	8.9
DROSE	*Drosophila serrata*	XP_020803791.1	8.7
DROVI	*Drosophila virilis*	XP_002057023.1	8.8
DROGR	*Drosophila grimshawi*	XP_001992161.1	8.5
DROBP	*Drosophila bipectinata*	XP_017090607.1	8.8
DRONO	*Drosophila novamexicana*	XP_030568677.1	8.8
DROTA	*Drosophila takahashii*	XP_017009012.1	8.7
DROER	*Drosophila erecta*	XP_001976950.1	8.8
DROAN	*Drosophila ananassae*	XP_014761224.1	8.8
LUCCU	*Lucilia cuprina*	XP_023296875.1	8.8
DROBI	*Drosophila biarmipes*	XP_016961519.1	8.7
DRORH	*Drosophila rhopaloa*	XP_016983377.1	8.8
DROSU	*Drosophila suzukii*	XP_016923010.1	8.9
STOCA	*Stomoxys calcitrans*	XP_013114326.1	8.4
DROKI	*Drosophila kikkawai*	XP_017030393.1	8.7
DROEU	*Drosophila eugracilis*	XP_017083376.1	8.7
SCALE	*Scaptodrosophila lebanonensis*	XP_030378843.1	8.9
DROBU	*Drosophila busckii*	XP_017851482.1	8.5
DROMI	*Drosophila miranda*	XP_017134982.1	8.7
DROPS	*Drosophila pseudoobscura*	XP_001355409.1	8.7
DROOB	*Drosophila obscura*	XP_022227514.1	8.7
DRONA	*Drosophila navojoa*	XP_017965737.1	8.6
DROHY	*Drosophila hydei*	XP_023162016.1	8.2
DROPE	*Drosophila persimilis*	XP_002027085.1	8.7
DRONV	*Drosophila novamexicana*	XP_030568678.1	7.4
DROMO	*Drosophila mojavensis*	XP_002010046.1	8.5
DROGU	*Drosophila guanche*	A0A3B0KTY9_DROGU	8.7
ZEUCU	*Zeugodacus cucurbitae*	A0A0A1WFG6_ZEUCU	8.8
CERCA	*Ceratitis capitata*	W8CBQ7_CERCA	8.1
MUSDO	*Musca domestica*	A0A1I8M261_MUSDO	8.0
BACDO	*Bactrocera dorsalis*	A0A034W4B0_BACDO	8.8

BACDO, CERCA, MUSDO and ZEUCU entries were identified using Psi-Blast and are included as Dhd-family members based on the conservation of Arg/Lys residues characteristic of Dhd proteins. The identity and similarity to Dhd and Trx-2 DROME proteins are indicated to highlight that similarity cannot be exclusively used to classify Dhd proteins. Anopheles sequences (*Nematocera*) were also retrieved using Psi-Blast but in this case these proteins are considered to be Trx-2 proteins because the similarity/identity is observed for residues conserved between Trx-2 and Dhd but not for additional Arg/Lys residues only present in Dhd proteins. Calculated isoelectric points (Kozlowski, 2016) are included to highlight that Dhd proteins have IP values larger than 7 and very often larger than 8. A sequence comparison of these divergent sequences is included as Supplementary Figure1F.

		**Dhd (IP 8.7)**		**Trx-2 (IP 4.6)**		
**Acronym**	**Species**	**Identity**	**Similarity**	**Identity**	**Similarity**	**IP**
BACDO	*Bactrocera dorsalis*	49.8%	83.0%	51.0%	73.0%	8.8
CERCA	*Ceratitis capitata*	54.0%	72.0%	48.0%	75.0%	8.1
MUSDO	*Musca domestica*	52.0%	88.2%	46.0%	72.0%	8.0
ZEUCU	*Zeugodacus cucurbitae*	43.0%	71.0%	60.0%	75.0%	8.8
9DIPT	*Anopheles braziliensis*	40.8%	82.5%	61.0%	80.0%	4.8
ANOFN	*Anopheles funestus*	45.0%	84.7%	56.0%	78.0%	4.6

Table S3 TrxT protein acronyms and entries used in the alignments.

Acronym	Species	RefSeq
DROME	*Drosophila melanogaster*	Q8IFW4.1
DROSE	*Drosophila sechellia*	XP_002036934.1
DROSI	*Drosophila simulans*	XP_016038140.1
DROMA	*Drosophila mauritiana*	XP_033171201.1
DROBI	*Drosophila biarmipes*	XP_016962073.1
DROSU	*Drosophila suzukii*	XP_016923019.1
DROER	*Drosophila erecta*	XP_001976949.1
DROYA	*Drosophila yakuba*	XP_002100028.1
DROTA	*Drosophila takahashii*	XP_017009366.1
DROAN	*Drosophila ananassae*	XP_001964015.1
DROBP	*Drosophila bipectinata*	XP_017102470.1
DROEU	*Drosophila eugracilis*	XP_017065989.1
SCALE	*Scaptodrosophila lebanonensis*	XP_030378842.1
DROWI	*Drosophila willistoni*	XP_002071507.1
DROEL	*Drosophila elegans*	XP_017129970.1
DROPE	*Drosophila persimilis*	XP_002021871.1
DROMI	*Drosophila miranda*	XP_017134981.1
DROPS	*Drosophila pseudoobscura*	XP_001355410.2
DROOB	*Drosophila obscura*	XP_022227513.1
DROVI	*Drosophila virilis*	XP_002057022.1
DROBU	*Drosophila busckii*	XP_017850961.1
DRONO	*Drosophila novamexicana*	XP_030568676.1
DROHY	*Drosophila hydei*	XP_023162014.1
DROGU	*Drosophila guanche*	SPP89275.1
DROMO	*Drosophila mojavensis*	XP_002011512.1
DRONA	*Drosophila navojoa*	XP_017963337.1
DROAR	*Drosophila arizonae*	XP_017870321.1
DROFI	*Drosophila ficusphila*	XP_017050179.1
BACDO	*Bactrocera dorsalis*	XP_011199125.1
ZEUCU	*Zeugodacus cucurbitae*	XP_011180011.1
BACOL	*Bactrocera oleae*	XP_014095633.1
RHAZE	*Rhagoletis zephyria*	XP_017484976.1
CERCA	*Ceratitis capitata*	XP_004521006.1

Table S4 Tm values at different pH and buffers

	Citrate	Acetate	Cacodylate	BIS-TRIS	ADA	MOPS	Tris	BICINE	AMPD
pH	4.5	4.9	6.1	6.6	7.0	7.4	8.5	8.7	9.3
TrxT	52 ±0.2	62±0.6	54±0.3	65±0.2	68±0.1	69±0.1	68±0.1	65±0.1	54±0.2
Dhd	85±0.1	75±0.1	73±0.2	68±0.3	73±0.1	63±0.1	60±0.1	61±0.1	62±0.6

Citrate: Sodium Citrate tribasic di-hydrate, acetate: sodium acetate trihydrate, cacodylate: sodium cacodylate trihydrate, ADA, N-(2-acetamido) iminodiacetic acid, MOPS (3-(N-morpholino) propanesulfonic acid), Tris: trisaminomethane, BICINE: N,N-Bis(2-hydroxyethyl)glycine, AMPD (2-amino-2-methyl-1,3-propanediol)

Supplementary Figure 1

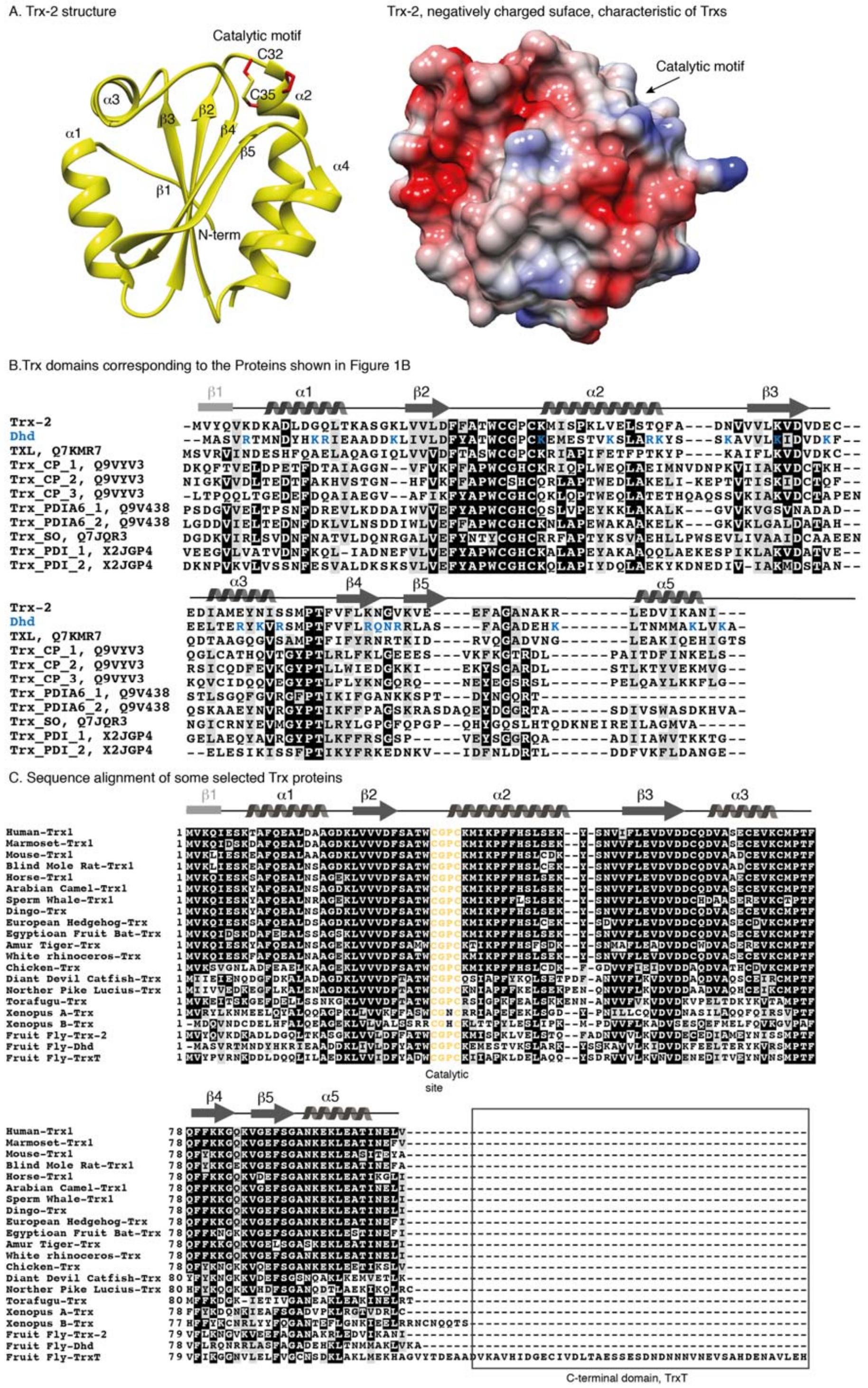

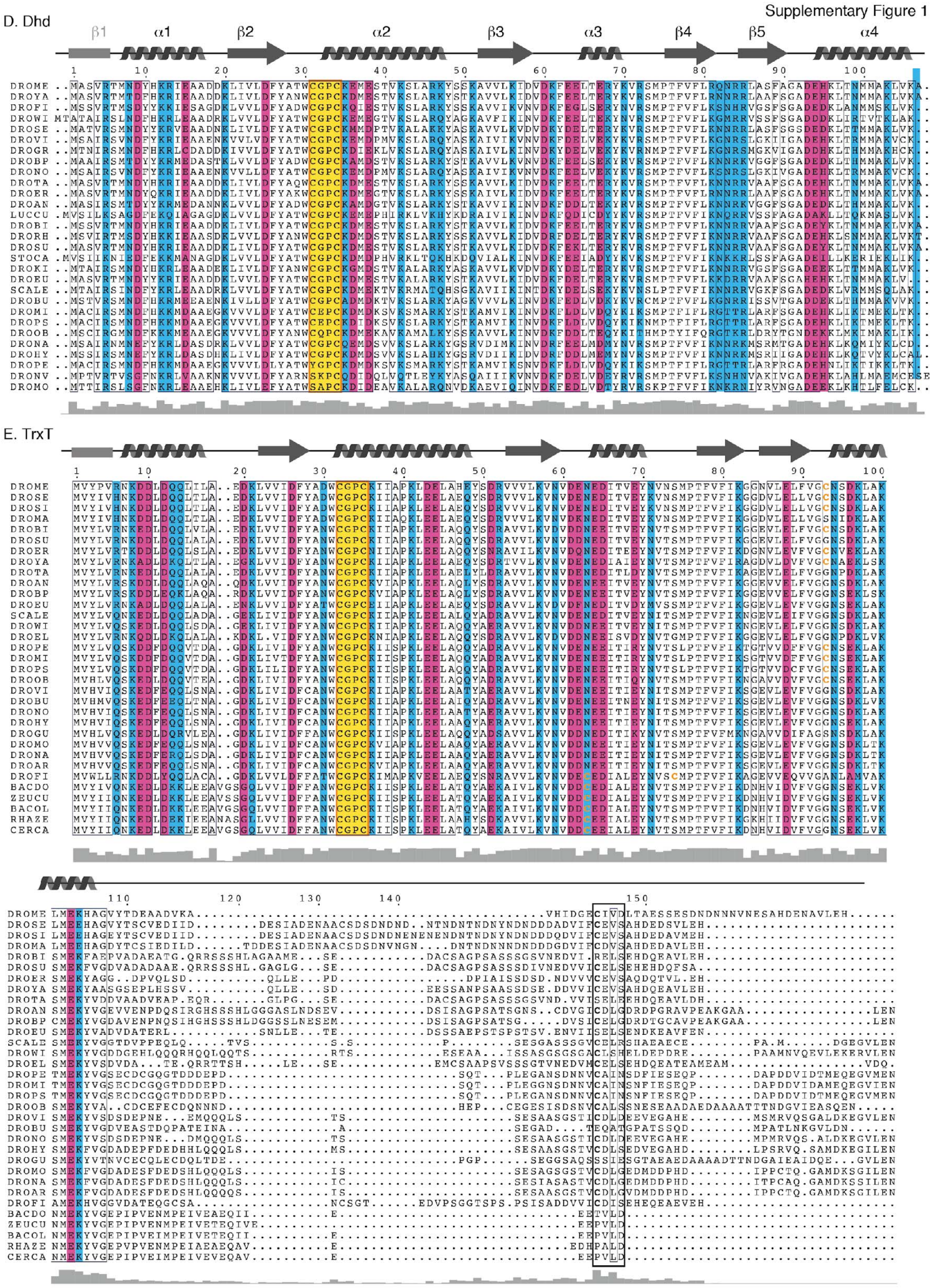
Supplementary Figure 1
D. Dhd
β1
α1
β2
α2
β3
α3
β4
β5
α4
E. TrxT

F. Dhd and Trx-2 proteins

Supplementary Figure 1

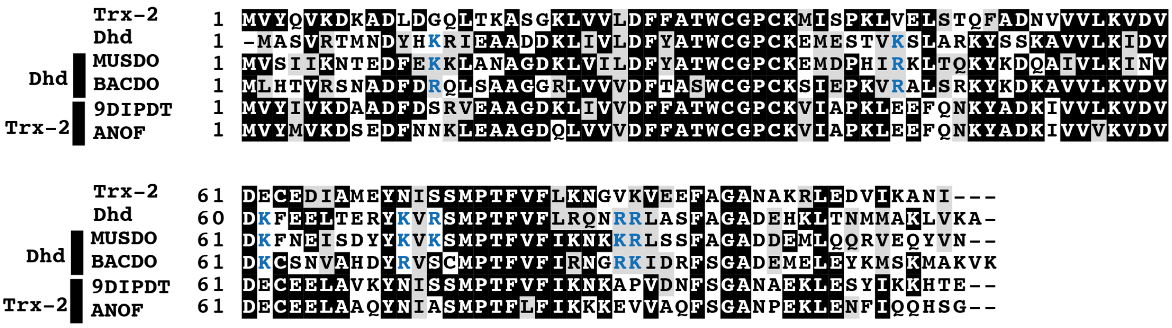

Figure S1 Thioredoxin sequence comparison. **A.** Ribbon diagram of the Trx-2 structure in its oxidized form (PDB:1XWA). All secondary structure elements are labeled. Molecular surface properties. Electrostatic potential of Trx-2, in which positively and negatively charged regions are shown in blue and red, respectively **B.** Trx domains corresponding to the Proteins shown in Figure 1B **C.** Sequence comparison of some selected vertebrate species and three *D. melanogaster* thioredoxins, (Tr-x2, TrxT and Dhd). RefSeq codes and species are indicated in Supplementary Table 1. The alignment was generated with Clustal Omega (EMBL-EBI) and the figure with BoxShade v3.21 (ExPASy). **D**. Extended version of the alignment of Dhd protein sequences shown in Figure 1C. The conservation level is indicated at the bottom of the alignment. Figure prepared with ESPript 3.0. **E**. Extended version of the alignment of TrxT protein sequences shown in Figure 1D. The conservation level is indicated at the bottom of the alignment. Figure prepared with ESPript 3.0. **F**. Comparison of divergent Dhd and Trx-2 sequences identified using Psi-Blast. Additional Lys and Arg residues present in Dhd but absents in TrxT are highlighted in blue.

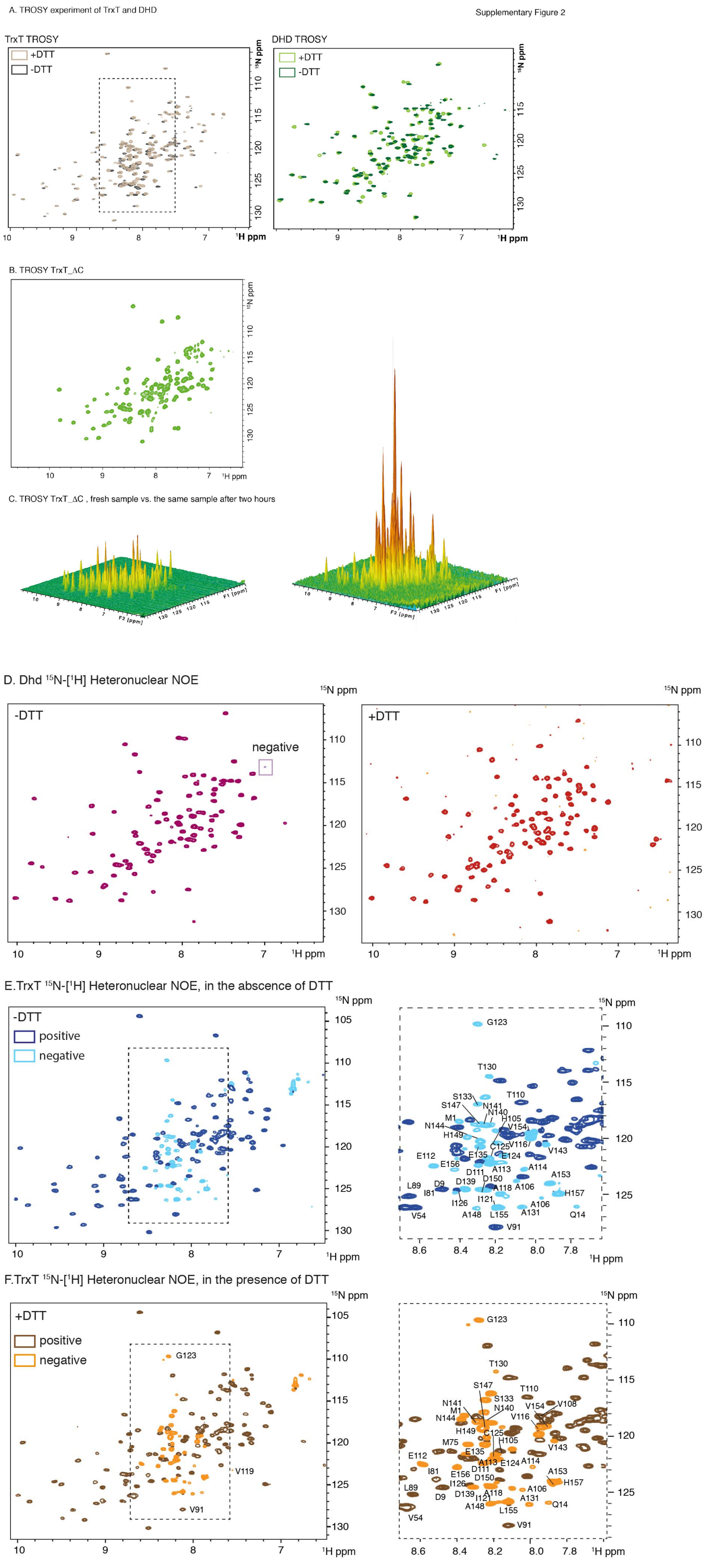

Figure S2 Structural characteristics of Dhd and TrxT by NMR and X-ray **A.** 2D ^{1}H, ^{15}N TROSY (Transverse relaxation optimized spectroscopy experiments) of TrxT and Dhd proteins, in the

presence or absence of DTT. Chemical shift variations observed upon addition of DTT are interpreted as the results of modifications in the redox state of the proteins. The region containing the residues assigned to the C-terminal domain is indicated with a box. **B.** 2D ^{1}H-^{15}N TROSY of TrxT-ΔC domain (aa1-111) indicating well-dispersed amides. **C.** 2D ^{1}H-^{15}N TROSY data of the TrxT-ΔC construct immediately after purification (left) and after two hours (right). The amide dispersion is maintained but the intensities are drastically changed, with more intense peaks populating the region characteristic of unfolded proteins. **D.** Same experiments as those shown in A and B for Dhd. The fold is highly defined and negative peaks are not detected (with the exception of one side-chain). **E.** 2D ^{15}N-[^{1}H] heteronuclear NOEs (run as duplicates) indicating the positive and negative peaks of TrxT in the absence of DTT. Resonances corresponding to the C-terminal domain are labeled. A few corresponding to well-structured regions are also indicated for comparison. **F.** 2D ^{15}N-[^{1}H] heteronuclear NOEs (run as duplicates) in the presence of DTT. Some chemical shift variations are observed with respect to A, indicating the effect of DTT on the redox properties of the sample. In both cases, Cys125 displays negative NOEs, thereby indicating that its internal motion is not fully dependent on its redox state.

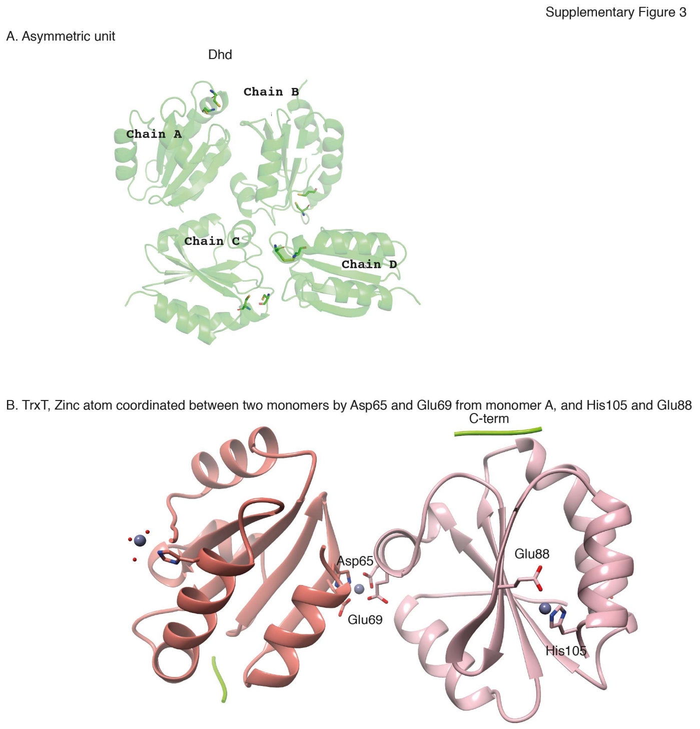

Figure S3 Flexible properties of the TrxT C-terminal domain. **A.** Cartoon representation of the asymmetric unit for the deadhead protein structure composed of four monomers. **B.** Cartoon representation of a symmetry-related dimer of the TrxT protein. Two monomers are engaged in a dimer interaction with symmetry-related neighbors through the coordination of a Zn atom. Residues involved in Zn interactions are labeled. The fragment of the C-terminal domain bound to Cys125 is shown in chartreuse. The rest of the C-terminal domain is not shown for simplicity.

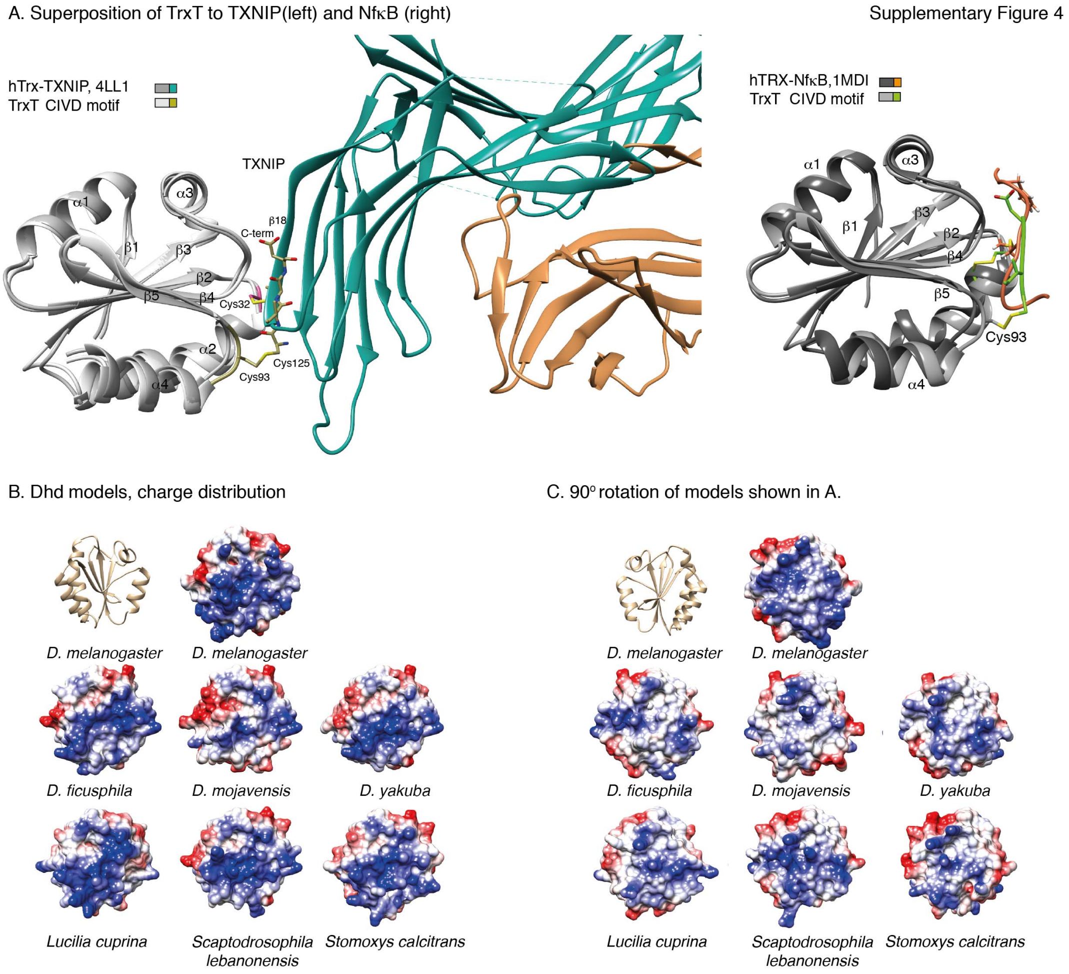

Figure S4 Charge distribution of Dhd models based on Dm Dhd structure. **A.** Comparison of the overall structure of the TrxT and hTRX in complex with TXNIP (left, PDB:4LL1) and hTRX in complex with NFκB (right, PDB:1MDI), using human Trx for the fitting. The C-term CIVD motif of TrxT (shown in chartreuse) occupies the same place as the Trx partners in the human Trx complex structures. **B.** Structure and charge distribution of Dm Dhd (top) and of six additional Dhd sequences oriented like Dm Dhd. **C.** A 90-degree rotation of the surfaces displaying the other side of the molecule.
